# Collaborative elicitation process for sustainable manufacturing: A novel evolution model of green technology innovation path selection of manufacturing enterprises under environmental regulation

**DOI:** 10.1371/journal.pone.0266169

**Published:** 2022-06-10

**Authors:** Manman Wang, Shi Yin, Shuai Lian

**Affiliations:** 1 College of Economics and Management, Zhengzhou University of Light Industry, Zhengzhou, China; 2 College of Economics and Management, Hebei Agricultural University, Baoding, China; 3 Zhengzhou Institute of Mechanical and Electrical Engineering, Zhengzhou, China; University of Akron, UNITED STATES

## Abstract

Due to promote manufacturing enterprises to carry out green technology innovation practice smoothly, achieve the goal of energy conservation and emission reduction, and win green competitive advantage, this paper first divides the green technology innovation path into two types, namely internal independent R&D of green technology (IIGT) and external green technology introduction (EGTI), and analyzes the operation mechanism of these two types of paths. Secondly, a two-agent game model of different types of environmental regulation tools on the choice of green technology innovation path of manufacturing enterprises is constructed. To be sure, the manufacturing enterprises include the leader enterprise A and the follower enterprise B. It is assumed that the two groups of manufacturing enterprises produce the same products or provide the same services in the natural state without considering other influencing factors. Finally, stability analysis and numerical simulation are employed to compare and analyze the heterogeneous effects of different environmental regulation tools on the path selection of green technology innovation in manufacturing enterprises.The simulation shows that when the government adopts or does not adopt environmental regulation means, the system, leader enterprise A and follower enterprise B will eventually choose the path of IIGT or EGTI respectively after a long-term evolution process. However, the effects of subsidy for green technology innovation and carbon tax rate on the path selection of green technology innovation are different among the three parties. Specifically, when the government adopts the means of subsidy for green technology innovation, the leader enterprise A will actively choose the the path of IIGT earlier than the follower enterprise B. On the contrary, when the government adopts the means of carbon tax, the leader enterprise A will actively choose the the path of IIGT later than the follower enterprise B. The research of this paper is helpful to explore the green and sustainable development mode of China’s manufacturing industry under the dual constraints of environment and resources, and provides decision support for the relevant national departments to make relevant policies.

## 1. Introduction

**Fig 1:**
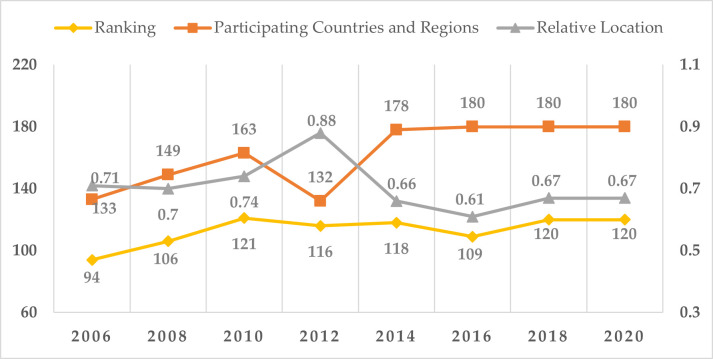
China’s EPI ranking changes.

In recent years, environmental problems such as global warming, oil crisis, water pollution and haze have been worsening [1–5]. Mainly the environmental problems are associated with industrial and agricultural sectors [6–8]. At present, China’s environmental quality problem is very serious. Based on the Environmental Performance Index (EPI) report jointly released by Yale University and Columbia University in the United States, it depicts the change of China’s EPI ranking from 2006 to 2020 in Fig 1 and can be found that China has always been at the bottom of the ranking of participating countries and regions. Therefore, China faces great international pressure in the process of global environmental governance, and the environmental problem has been attached great importance by the government of China, and relevant policies have been introduced one after another. The CPC Central Committee and The State Council issued the Opinions on Accelerating the Construction of ecological Civilization in 2015, which specifically pointed out that “we must build an industrial structure with high science and technology content, low resource consumption and less environmental pollution, accelerate the greening of production methods and significantly improve the greening of the economy”. The communique of the fifth Plenary Session of the 19th CPC Central Committee in 2020 stressed the need to accelerate green and low-carbon development, continuously improve the quality of the environment, enhance the quality and stability of the ecosystem, and comprehensively improve the efficiency of resource utilization. The government of China has come up with the “double carbon” targets and pledged to achieve a green and low-carbon development goal of reducing carbon dioxide emissions per unit of GDP by 60% to 65% from 2005 levels by 2030.

Manufacturing activities are regarded as a major source of pollution [9]. Due to save energy and reduce emission and enhance enterprise competitiveness, more and more manufacturing enterprises begin to pay attention to green technology innovation. Compared with traditional technology innovation which emphasizes too much on economic benefits, green technology innovation has the double externalities. It pays attention to the economic development of the whole society, emphasizes on reducing the environmental burden of enterprises by introducing new systems, products and processes, and can bring economic value and competitive advantage to enterprises [10]. In practice, green innovation has the characteristics of high cost, long cycle and high risk [11, 12]. At present, the independent green innovation capability of Chinese manufacturing enterprises is weak, and many enterprises have negative attitudes due to the high barriers to green technology innovation and high R&D cost, which slows down the speed of enterprise green technology innovation and leads to the worsening of environmental pollution. The research shows that the government is the external environmental manager of the country, and its environmental regulation means will indirectly affect the green technology innovation behavior of enterprises. From the current international practice, subsidies for green technology innovation and carbon taxes are two common means of carbon emission reduction. The green innovation incentive fees compensated by the government for environmental pollution and the carbon taxes collected by the government will indirectly affect the final profits of enterprises and promote them to carry out green innovation. The correct path of green technology innovation can promote the development of green innovation, realize the virtuous circle between green economic growth and ecological environment protection of manufacturing industry, and improve its ability of green technology innovation. Therefore, under the background of green development, it is of great significance to explore the impact of environmental regulation on the green technology innovation path of manufacturing enterprises and form a benign interaction between the government and manufacturing enterprises, which is conducive to improving the green technology innovation ability of enterprises and realizing the sustainable development of society.

However, most of the previous researches tend to study the game relationship between government and enterprises, and pay less attention to the game relationship between enterprises. This paper aims to analyze the process and mechanism of environmental regulation on the selection of manufacturing enterprises’ green technology innovation path. How does the choice of green technology innovation path among individual manufacturing enterprises affect each other? What is the heterogeneity of different types of environmental regulation tools on manufacturing enterprises’ choice of green technology innovation path? These are the main problems to be solved in this paper. Combined with China’s green practice, based on the different connotation of innovation subject and new technology source, the green technology innovation path of Chinese manufacturing enterprises is divided into two types, namely internal independent R&D of green technology (IIGT) and external green technology introduction (EGTI). From this perspective, this paper builds a duopoly model of the leader enterprise and the follower enterprise. Furthermore, theoretical analysis and numerical simulation are conducted to analyze the co-evolution process of game players and the influence of different types of environmental regulation tools on the path selection process of green technology innovation in manufacturing enterprises. The purpose of this paper is to provide decision-making basis for encouraging enterprises to carry out green innovation practice and to ensure that the government achieves the goal of energy conservation and emission reduction.

The remaining parts of this paper are organized as follows: Section 2 briefly provides a literature review; The operation mechanism of green technology innovation path and constructs the theoretical research framework are presented in Section 3; Section 4 displays a duopoly model and evolutionary stability analysis of the leader enterprise and the follower enterprise; Section 5 depicts the results of theoretical analysis and numerical simulation; and Section 6 summarizes the conclusions and some major policy implications.

## 2. Literature review

### 2.1. Green technology innovation path

#### 2.1.1 The connotation of green technology innovation path

Path is a geographical term used to describe a route to a destination. At the beginning, professor Xu introduced the path into the research of enterprise technology development. He proposed that technology path refers to the channel between different strategic plans and the attraction of future opportunities to achieve strategic goals, which depends on the advantages accumulated by enterprises and the possibilities of opportunities [13]. Zhang put forward the concept of technology innovation path for the first time and applied it to the research of national technological innovation strategy selection under different social environment and institutional background [14]. Subsequently, many scholars have carried on the extension research of technology innovation path from the perspectives of innovation mode selection and orientation [15–17], innovation path dependence [18–20] and breakthrough of enterprise innovation strategy [21, 22].

There is no unified definition of technology innovation path in academic circles, and scholars have also expounded the connotation of enterprise technology innovation path from different perspectives. For example, from the perspective of concrete practice, Abernathy and Utterback defined technology innovation path as the track formed by the development of the innovation activities of mature enterprises with industrial technological standards along the dominant design direction and proposed the classic model A-U model in the field of technological innovation [23]. Xie et al. started from the direction of technology innovation and believed that technology innovation path refers to various alternative and innovation-oriented technological development plans that enterprises have to realize strategic goals, which points out strategic directions for enterprises to carry out technology innovation activities [24]. Wang studied the mechanism and unlocking mode of technological innovation path, and believed that the path of technological innovation includes improving people’s innovation ability, improving external environment, implementing benefit induction and external impact [25]. Wang analyzed the significance of technological innovation path to technological innovation from the perspective of evolutionary economics, and held that the technological innovation path of enterprises was limited by the technology conversion cost and the complementarity of industries [26]. In contrast to green technology innovation path which is less studied, the connotation of green process innovation path has a certain research basis. For example, Astino proposed that green process innovation path should be studied from design, process, production, circulation and other links [27]. Urmila and Yogendra considered that green process design includes design decisions in the period of chemical and material selection as well as prior decisions in management and planning [28]. Mroczkowski believed that the green process innovation path of the home appliance manufacturing refers to the combination of innovation modes, methods and methods suitable for its development in the implementation of green process innovation in the home appliance manufacturing according to the actual situation of its own ecological nich [29].

#### 2.1.2 The division of green technology innovation path

Since Joseph Schumpeter put forward the innovation theory, scholars have divided the technological innovation path and green technology innovation path from different perspectives based on research objectives. In terms of innovation intensity, technology innovation can be divided into progressive technology innovation and breakthrough technology innovation. The former refered to the incremental and continuous innovation caused by the improvement of existing technology. The latter refered to innovations based on breakthrough technologies, such as those that were not on the performance improvement track for mainstream customers [30]. From the perspective of innovation object, innovation path can be divided into product innovation path and process innovation path. The former refers to the path of creating a new product or innovating the functionality of a new or old product. The latter refered to the adoption of new or significantly improved production methods, process equipment or auxiliary activities [31, 32]. Considering the relationship between innovation subject and innovation means, the innovation path includes independent innovation path, cooperative innovation path and imitation innovation path. Independent innovation path refered to the process of realizing the value of new products on the basis of the unique core technology owned by enterprises with independent intellectual property rights. Cooperative innovation path was an activity in which each cooperative subject achieves the same goal by means of various forms of cooperation. Imitation innovation path was the innovation activity through imitation [33, 34]. Chesbrough further proposed that technology innovation can be divided into closed innovation and open innovation. The former refered to the integrated innovation in which the enterprise controled the whole process from the idea to the launch of a new product. The latter refered to opening up the traditional closed innovation mode and introducing external innovation capability [35]. Similarly scholars have studied the division of green technology innovation path. Bi and Shen summarized three main knowledge spillover transmission paths of manufacturing green innovation system, including knowledge spillover based on green R&D cooperation, green technology transformation and green product flow [36]. Xu et al. divided the green innovation mode of manufacturing enterprises into progressive green innovation and breakthrough green innovation [37]. Bi et al. divided the breakthrough innovation of low-carbon technology in manufacturing into breakthrough product innovation, breakthrough process innovation and breakthrough service innovation [38]. Shi and Tian proposed three alternative paths of green process innovation in home appliance manufacturing, including green process introduction and imitation innovation, green process collaboration and cooperative innovation, and green process creation and independent innovation [39].

### 2.2. Environmental regulation and green technology innovation path

There are many literatures about environmental regulation and green technology innovation.However, the research about environmental regulation on the choice of green technology innovation path is insufficient. In addition, the current research mainly focuses on the empirical study of environmental regulation on green technology innovation. In view of the relationship between environmental regulation and green technology innovation, scholars have undertaken significant research mainly from the macroscopic and microscopic aspects. The macroscopic research focuses on utilizing the empirical method to verify the effect of environmental regulation on green technology innovation. There are three main arguments for this kind of research. The first view is that environmental regulation can positively affect green technology innovation. For example, Cole et al. proposed that compared with countries with lax regulatory policies, countries with strict regulatory laws have a higher probability of innovation, and environmental regulation has a positive impact on technology innovation [40]. Deng et al. considered that the environmental regulation effect brought by the “energy saving and low carbon” policy has a significant effect in promoting the green innovation ability of enterprises [41]. The second view is that environmental regulation can negatively affect green technology innovation. For example, Testa et al. believed that environmental regulations seriously hinder the implementation of technological innovation due to increasing the cost burden of enterprises [42]. Yu and Hu took China’s heavily polluting manufacturing industry as an example and empirically found that environmental regulation has a negative impact on technological innovation capability in all phases [43]. The third view is that there is not a single linear relationship between environmental regulation and green technology innovation. For example, Wang and Shen empirically concluded that the relationship between environmental regulation intensity and environmental efficiency was u-shaped and there were three thresholds [44]. The microscopic research tends to compare the heterogeneity influence of environmental regulation tools on green technology innovation. For example, Wang believed that command control and market regulation are the most effective pollution control policy tools in China at present, and the regulatory effect of public participation and voluntary regulation is weak [45]. Sun and Liu theoretically analyzed the relationship between environmental regulation, clean technology innovation and industrial green transformation. The empirical results showed that environmental regulation has a significant threshold effect on industrial green transformation, and only when environmental regulation is lower than a certain threshold value can industrial green transformation be promoted [46].

In general, there are few studies on the green technology innovation path of manufacturing enterprises and the impact of environmental regulation on the green technology innovation path. In addition, most of the research methods are empirical and the research object is relatively single. In reality, green innovation is faced with a complex and changeable environment in which multiple actors participate. The interaction between manufacturing enterprises will have an important impact on their decision-making process. Different decision-making subjects will make judgments based on the decision-making behavior of other subjects, and there is a game relationship between decision-making bodies. Therefore, the evolutionary game theory with bounded rationality hypothesis and incomplete information advantage is suitable for the study of dynamic decision-making in the process of competition. At the same time, the research on technology innovation path focuses mostly on the qualitative research of technological innovation mode, direction and methods, with a lack of systematic and quantitative research. In addition, scholars have done little research on the path selection of green technology innovation. Relevant research shows that the environmental regulation tools of government, such as green innovation subsidy and carbon tax, are the main driving factors for enterprises to adopt green behaviors. However, most of the existing literatures carry out theoretical analysis on the economic impact and emission reduction effect of subsidy for green innovation and carbon tax respectively. Few scholars place the two regulatory tools in a unified research framework for comparative research, and most of them ignore the differences in the impact mechanism of different environmental regulation tools on the path selection of green technology innovation. Besides, there are few quantitative studies and simulation analysis based on evolutionary game theory. Hence, it is necessary to explore the evolutionary mechanism of green technology innovation path selection of manufacturing enterprises under the effect of different types of environmental regulation tools.

In the face of the deteriorating ecological environment in China, whether environmental regulation can promote the green transformation of manufacturing industry through technology introduction and independent innovation is a question worthy of further discussion. We make a contribution to the studies discussed above in several ways. First, we analyze operation mechanism of green technology innovation path and the heterogeneous influence mechanism of environmental regulation on the path of IIGT and EGTI. Second, we construct a two-agent game model of environmental regulation on the green technology innovation path selection of manufacturing enterprises. To this end, the impact effect of the parameter change of the subsidy rate and carbon tax rate on the evolution path and speed of green technology innovation path selection of the system, leader enterprise A and follower enterprise B are simulated.

## 3. Theoretical framework

### 3.1. Operation mechanism of the green technology innovation path of manufacturing enterprises

China’s national innovation strategy in the new period includes independent innovation guided by original innovation and technology introduction based on digestion and absorption. On the one hand, manufacturing enterprises with advanced technology have more R&D personnel and capital investment in green technology, and pursue the leading advantages in technology through independent innovation. On the other hand, manufacturing enterprises with backward technology are characterized by weak knowledge resources, technical resources and human resources. When they are not capable of independent innovation, they can completely introduce and imitate the mature technology through technology introduction. Under the pressure of environment and cost, it is necessary to introduce energy-saving and environment-friendly production technology from outside to offset external costs such as environmental expenditure and reduce environmental consumption while improving production efficiency [47].

**Fig 2:**
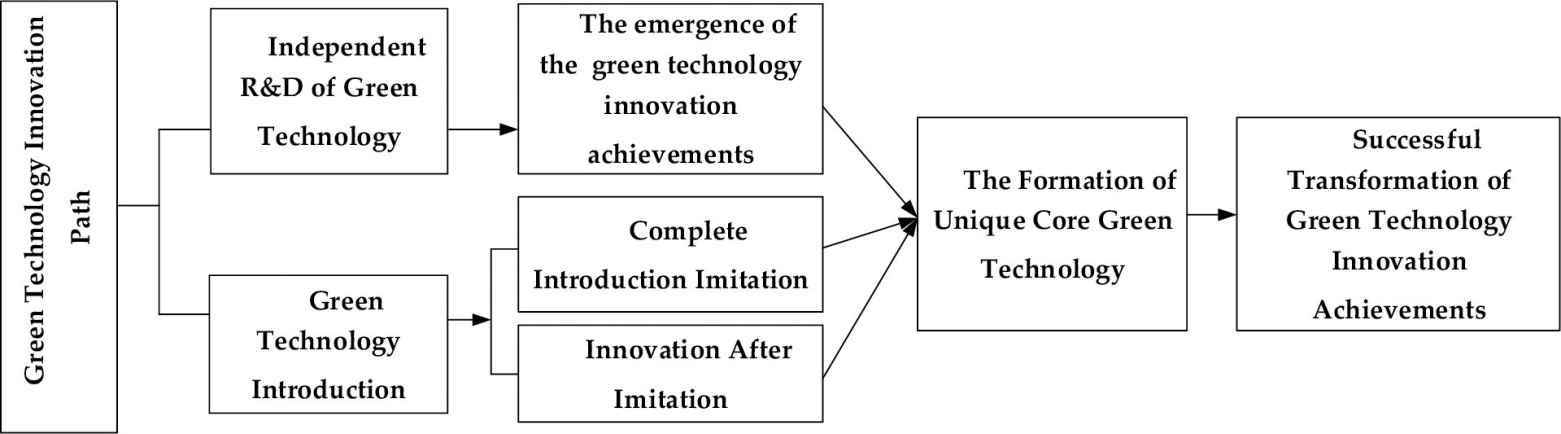
The division of green technology innovation path.

Based on the above review, this paper holds that the green technology innovation path of manufacturing enterprises refers to the dynamic development process from the internal independent R&D of green technology (IIGT) and external green technology introduction (EGTI) to the application of green new technology for the purpose of achieving green technology innovation [48]. Taking into account China’s national conditions, based on the different connotation of innovation subject and new technology source, the green technology innovation path of China’s manufacturing enterprises is divided into two kinds of green technology innovation path including IIGT and EGTI. Thereinto, the path of IIGT is a typical integration of research and development mode. The definition is that manufacturing enterprises (especially leading enterprises) achieve green technology breakthroughs through research and development, such as developing green new products and providing green technical support, and rely on the green technology advantages to obtain market monopoly position and excess profits so as to maintain the leading position in the industry [49]. The path of EGTI refers to the mode in which manufacturing enterprises directly obtain advanced and applicable green technology from other enterprises, research institutes and institutions at home or abroad in a certain way to realize the improvement of green technology innovation ability [50]. It depicts the operating mechanism of two types of green technology innovation paths in Fig 2.

#### 3.1.1 The connotation of green technology innovation path

Under this path, based on their own green technology R&D capabilities, manufacturing enterprises overcome further R&D difficulties through independent research, seek more energy saving and environmental protection production processes, form achievements of green technology innovation, and obtain economic benefits while protecting the ecological environment. Specifically, the success of IIGT will bring the dual monopoly advantages of green technology market and green product market. Even if the enterprise is not capable of industrialization and marketization, it can also obtain the corresponding benefit value through its huge future income. When the achievements of green technology innovation are successfully transformed and have not been imitated by other competitors in the same industry, the market monopoly advantage of green new products will bring huge consumer surplus value to the enterprise. The size of the market share determines the position of the enterprise in the industry. When an enterprise’s green products have become the object of imitation by other followers, the enterprise has become the industry leader. The high returns brought by the dual monopoly of green technology market and green product market will be fed back to the enterprises themselves and encourage them to continue to choose the path of IIGT. In this virtuous circle, manufacturers can seize the technological and economic competitive commanding heights. The green technology that enter the market ahead have a strong ability to resist market risks, and manufacturing enterprises can rely on them to continuously create green technologies in line with sustainable development. From the technical basis requirements of independent innovation, the path of IIGT is more suitable for the leading enterprises with industrial competitiveness [51].

#### 3.1.2 The operating mechanism of green technology introduction

The path of EGTI includes complete introduction and innovation after imitation. The path of complete introduction refers to the imitation of the green technology that first enters the market. Manufacturing enterprises acquire the leading advanced green technology in the industry by means of introducing or purchasing green technology, and achieve the standard of imitated green technology through digestion and absorption. The connotation of innovation after imitation is that manufacturing enterprises further create green technology with strong competitiveness by taking the lead in local innovation after mastering the imitated green technology. When a manufacturing enterprise has a low technological level and is not capable of independent innovation, it can completely introduce and imitate mature technologies through technology introduction to form products that can produce market economic benefits in a short period of time, and occupy the market and gain profits with the help of the marketing ability of the enterprise. Green technologies and products produced by the complete introduction are more likely to follow the market. Although manufacturing enterprises have a foothold in the market after complete introduction, they still lack core technologies and need to maintain their own development through innovation after introduction, which can also significantly reduce the sunk costs of indigenous innovation. When the achievements of green technology innovation produced by EGTI have not been widely used at home and abroad, the path of EGTI can produce monopoly advantages of green technologies and products similar to IIGT. In addition, compared with IIGT, EGTI takes less time and cost, and the uncertainty of innovation is smaller. This path enables manufacturing enterprises to achieve technological catch-up without relying on high green R&D investment, which ultimately improves the competitiveness of manufacturing enterprises. Manufacturing enterprises that adopt the path of EGTI generally have poor resource accumulation of green technology, and the talents and technologies required for green technology innovation are mainly obtained from outside. They can enhance their capacity of green technology innovation through technology purchase, technological transformation and external R&D personnel support [52].

### 3.2. Mechanism analysis of environmental regulation on green technology innovation path selection of manufacturing enterprises

The occurrence of different green technology innovation paths needs to be established under certain preconditions. The process from the emergence of green technology innovation behavior to the emergence of green technology innovation achievements or market products is also affected by the government’s environmental regulation policies. Specifically, in the early stage of environmental regulation implementation, enterprises are more willing to choose the technology import mode with lower cost to offset the pollution control cost caused by environmental regulation. However, with the narrowing of the gap between manufacturing enterprises and the increasing intensity of environmental regulation, the higher difficulty and cost of technology introduction will gradually inhibit the process of technology introduction. In particular, the high complexity of green technology makes it difficult and costly to introduce. It is impossible to improve the level of green technology through technology introduction, and the space of technology introduction is becoming saturated and the difficulty of technology introduction is gradually increasing [53]. Therefore, it is no longer sustainable for enterprises to rely on technology introduction to promote technological progress for a long time, and they begin to turn to independent innovation [54].

The environmental regulation measures taken by the government can be divided into subsidies for green innovation and carbon tax. Among them, subsidies for green innovation refer to a certain proportion of the total input of green technology innovation adopted by manufacturing enterprises. The government implements comprehensive subsidy policies according to the specific situation, which is to promote more enterprises to engage in green technology innovation activities and give full play to the environmental protection role of green products. For example, Garcia believed that government subsidies reduce fixed costs and relieve capital constraints and financing pressure of enterprises in the form of capital injection [55]. Wu et al. found that government subsidies can promote corporate innovation input of enterprises and help enterprises obtain resources and support from stakeholders in strategic emerging industries, which can directly accelerate the innovation output of enterprises [56]. Besides, the carbon tax is based on the carbon emissions of manufacturing enterprises, and the tax rate is multiplied by the carbon tax. This is a kind of environmental regulation measure prevailing internationally under the background of green and low-carbon economy, which has been widely concerned by scholars at home and abroad. The government has induced enterprises to adopt green technologies by raising the carbon tax rate [57]. For example, Meltzer took the United States as an example to study the impact of carbon tax on low-carbon technology innovation, and the research results showed that carbon tax was an important way to promote green technology innovation [58]. Meng and Han believed that innovation input subsidy or carbon emission trading policy alone has a poor incentive effect on enterprises’ low-carbon technology innovation behavior, but only when combined with carbon tax system can it have an effective incentive effect [59]. The essence of both of them is to solve the problem of how to actively guide enterprises to implement green production under the premise of maximizing enterprise interests.

**Fig 3:**
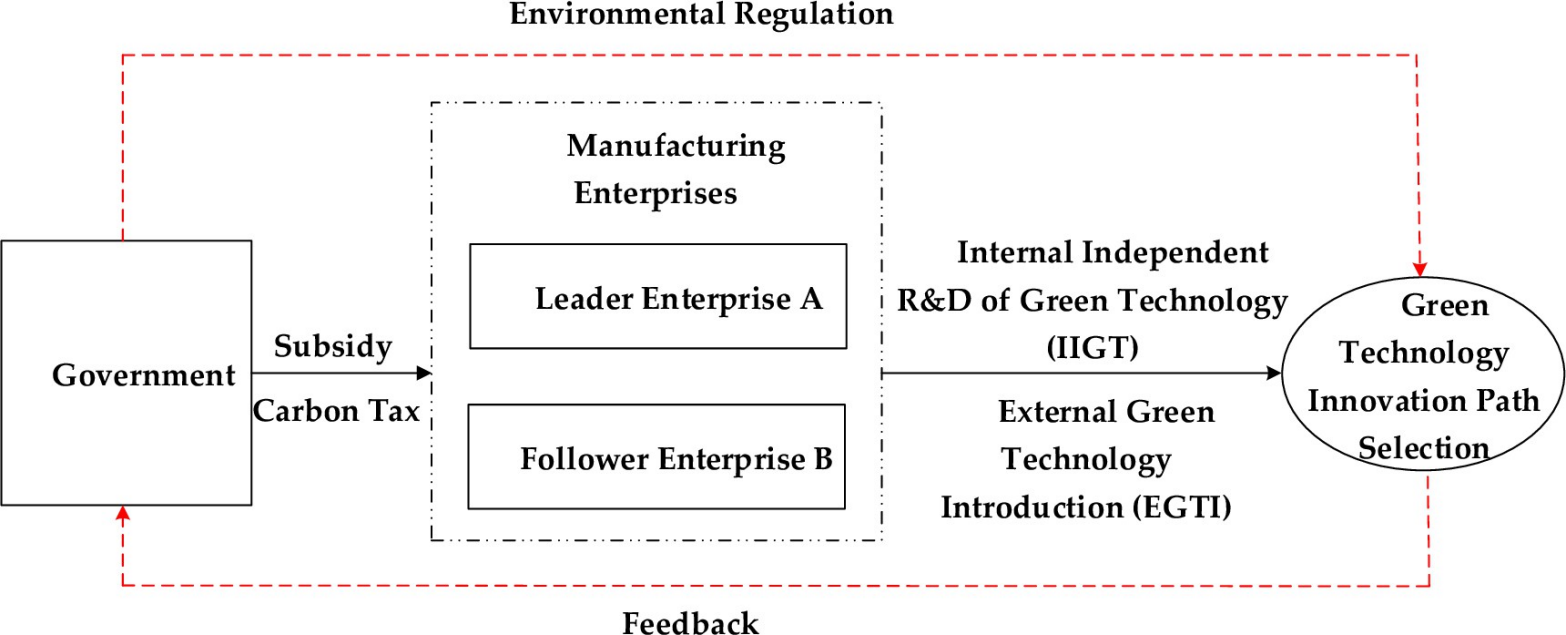
Illustrations of the game relationship among the government, the leader enterprise A and the follower enterprise B.

Evolutionary game emphasizes that path selection is the result of mutual behavior adjustment, which is consistent with the characteristics of path selection of green technology innovation. Path selection is a dynamic evolutionary process in which the behaviors of all parties adjust to each other under certain rules [60]. In the face of environmental regulation pressure, first of all, the managers of manufacturing enterprises which are as the decision-making body of enterprises’ green technology innovation behavior need to consider the input and income of enterprises when implementing the innovation. Secondly, the interaction between manufacturing enterprises will also have an important impact on the decision-making process. Different decision-making subjects will make judgment according to the decision-making behavior of other subjects, which reflects that there is game relationship among decision-making subjects. Finally, the path choice will change dynamically under the influence of the technology and market environment faced by manufacturing enterprises. Therefore, in order to scientifically depict the dynamic game process of green technology innovation path selection in manufacturing enterprises, this paper will use evolutionary game method to analyze the path selection problem. The theoretical framework of this paper is shown in Fig 3.

## 4. The evolutionary game model of green technology innovation path selection in manufacturing enterprises

On the whole, China is in the middle stage of industrialization dominated by manufacturing. China’s manufacturing enterprises have the foundation of green technology innovation to a certain extent and have certain abundant strength to carry out IIGT. However, due to the restrictions of property rights system and policy environment, some manufacturing enterprises are at a low level of green technology innovation ability and are more inclined to choose the path of EGTI depending on their own economic and technological basis. Therefore, this paper divides manufacturing enterprises with game behaviors of green technology innovation path selection into two categories: leaders with strong green technology innovation ability and followers with weak green technology innovation ability. Based on the condition of bounded rationality, stackelberg’s duopoly model is adopted in this section. To establish the evolutionary game model of green technology innovation path selection in manufacturing enterprises, the related assumptions and parameters are shown as follows:

### 4.1. Construction of hypothesis

**Hypothesis 1 (H1).** It is assumed that in a natural state without considering other influencing factors, a certain group of manufacturing enterprises producing homogeneous products or providing homogeneous services is divided into two types of monopoly groups, namely the leader enterprise A and the follower enterprise B respectively. When the leader enterprise A chooses its own output, it will know the response of follower enterprise B in advance and determine its output according to the response of follower enterprise B. All the members of this group have learning ability and slow learning speed, and they need to repeatedly learn and imitate other enterprises to form their own strategies. Members of the two groups were repeatedly selected for matching games in the course of evolutionary games.

**Hypothesis 2 (H2).** The strategy of green technology innovation path selection between the leader enterprise A and follower enterprise B are IIGT and EGTI, respectively. There are four strategy sets for green technology innovation path selection of two game players, which are (IIGT, IIGT), (IIGT, EGTI), (EGTI, IIGT) and (EGTI, EGTI). The corresponding game payoffs enterprise of two game players are (P_A11_, P_B11_), (P_A12_, P_B12_), (P_A21_, P_B21_) and (P_A22_, P_B22_).

**Hypothesis 3 (H3).** It is assumed that the green innovation costs of IIGT and EGTI of manufacturing enterprises are C_1_ and C_2_ respectively. Due to develop green and low-carbon economy, the government will encourage enterprises to implement green technology innovation and adopt two kinds of macro-control measures in line with the investment level of green innovation and carbon emissions of enterprises. On the one hand, the government gives subsidies to enterprises for green technology innovation input. On the other hand, the government imposes carbon tax on enterprises. The government subsidy rate for green innovation and the carbon tax rate will be set at α (0<α<1)and β (0<β<1). The carbon emissions of leader enterprise A after adopting the path of IIGT and EGTI will be set as K_1_ and K_2_. Similarly,The carbon emissions of follower enterprise B after adopting the path of IIGT and EGTI will be set as K_3_ and K_4_.

**Hypothesis 4 (H4).** Under the condition of bounded rationality, it is assumed that the probability of leader enterprise A choosing the path of IIGT and EGTI is x and (1-x). In a similar way, the probability of follower enterprise B choosing the path of IIGT and EGTI will be set as y and (1-y). x and y are functions of time t.

**Hypothesis 5 (H5).** In the absence of additional intervention, each player will spontaneously evolve through the natural law of “survival of the fittest” or the market law, and choose and adjust their own strategies according to the strategy selection of other group members and their relative adaptability in their own group.

### 4.2. The evolutionary game model

According to the above assumptions, the profits of leader enterprise A and follower enterprise B are recorded as U_A_(U_A1_, U_A2_) and U_B_(U_B1_, U_B2_) respectively for the selection strategies of different green technology innovation paths under the premise of government subsidies for green technology innovation and carbon tax. Both players aim at profit maximization. The total income and the payment matrices of the player in the game under the combination of the four strategies can be obtained. As shown in Table 1.

**Table 1:** Payment matrix of duopoly game under environmental regulation.

Game Player		Follower Enterprise B	
		IIGT	EGTI
**Leader Enterprise A**	**IIGT**	$\begin{array}{c} U_{A11}=P_{A11}-(1-\alpha )C_{1}=P_{A11}-C_{1}-\beta k_{1}\\ U_{B11}=P_{B11}-(1-\alpha )C_{2}=P_{B11}-C_{1}-\beta k_{3} \end{array}$	$\begin{array}{c} U_{A12}=P_{A12}-(1-\alpha )C_{1}=P_{A12}-C_{1}-\beta k_{1}\\ U_{B12}=P_{B12}-(1-\alpha )C_{2}=P_{B12}-C_{2}-\beta k_{4} \end{array}$
	**EGTI**	$\begin{array}{c} U_{A21}=P_{A21}-(1-\alpha )C_{2}=P_{A21}-C_{2}-\beta k_{2}\\ U_{B21}=P_{B21}-(1-\alpha )C_{1}=P_{A21}-C_{1}-\beta k_{3} \end{array}$	$\begin{array}{c} U_{A22}=P_{A22}-(1-\alpha )C_{2}=P_{A22}-C_{2}-\beta k_{2}\\ U_{B22}=P_{B22}-(1-\alpha )C_{2}=P_{B22}-C_{2}-\beta k_{4} \end{array}$

According to the payment matrix in Table 1, it can be calculated that the profit of leader enterprise A choosing the path of IIGT and EGTI are U_A1_ and U_A2_, and the average expected profit is $\bar{U_{A}}$.


$$U_{A1}=yU_{A11}+(1-y)U_{A12}$$ (1)

$$U_{A2}=yU_{A21}+(1-y)U_{A22}$$ (2)

$$\bar{U_{A}}=xU_{A1}+(1-x)U_{A2}$$ (3)

Similarly, it can be calculated that the profit of follower enterprise B choosing the path of IIGT and EGTI are U_B1_ and U_B2_, and the average expected profit is $\bar{U_{B}}$.


$$U_{B1}=xU_{B11}+(1-x)U_{B12}$$ (4)

$$U_{B2}=xU_{B12}+(1-x)U_{B22}$$ (5)

$$\bar{U_{B}}=yU_{B1}+(1-y)U_{B2}$$ (6)

## 5. Dynamic equilibrium analysis of green technology innovation path selection under different types of environmental regulation tools

### 5.1. Solution of equilibrium point under green innovation subsidy and carbon tax

Based on evolutionary game theory, it can be obtained from Eqs (1)–(6) that the replication dynamic equation of the leader enterprise A and follower enterprise B choosing the path of IIGT and EGTI under the tools of government subsidies for green technology innovation and carbon tax are *F*(*x*) and *F*(*y*).


$$\begin{array}{l} F(x)=dx/dt=x(U_{A1}-\bar{U_{A}})=x(1-x)(U_{A1}-U_{A2})=x(1-x)[(P_{A12}-P_{A22})-(1-\alpha )(C_{1}-C_{2})\\ \;+y(P_{A11}-P_{A21})-y(P_{A12}-P_{A22})] \end{array}$$ (7)

$$\begin{array}{l} F(y)=dy/dt=y(U_{B1}-\bar{U_{B}})=y(1-y)(U_{B1}-U_{B2})=y(1-y)[(P_{B12}-P_{B22})-(1-\alpha )(C_{1}-C_{2})\\ \;+x(P_{B11}-P_{B21})-x(P_{B12}-P_{B22})] \end{array}$$ (8)

In the process of green technology innovation path selection, the game players continue to evolve to a stable strategy. When the proportion of the two game players adopting the two types of green technology innovation path remains fixed, it reaches a stable state. At this time, the expression of the replication dynamic equation of both sides satisfies the following formula.


$$\{\begin{array}{c} F(x)=dx/dt=0\\ F(y)=dy/dt=0 \end{array}$$ (9)

According to the formula (9), there are five local equilibrium points in the evolutionary game model of the game players. They are O(0, 0), U(1, 0), V(0, 1), W(1, 1) and S(x*, y*) where

$$\{\begin{array}{l} x*=\frac{\left(1-\alpha )(C_{1}-C_{2})-(P_{B21}-P_{B22}\right)}{\left(P_{B11}-P_{B12})-(P_{B21}-P_{B22}\right)}=\frac{\beta (k_{3}-k_{4})+(C_{1}-C_{2})-(P_{B21}-P_{B22})}{\left(P_{B11}-P_{B12})-(P_{B21}-P_{B22}\right)}\\ y*=\frac{\left(1-\alpha )(C_{1}-C_{2})-(P_{A12}-P_{A22}\right)}{\left(P_{A11}-P_{A21})-(P_{A12}-P_{A22}\right)}=\frac{\beta (k_{1}-k_{2})+(C_{1}-C_{2})-(P_{A12}-P_{A22})}{\left(P_{A11}-P_{A21})-(P_{A12}-P_{A22}\right)} \end{array}$$ (10)

Then, the stable equilibrium point of the evolutionary system is obtained by analyzing the local stability of the Jacobian matrix of the replication dynamic equation, which can effectively judge the evolution direction of green technology innovation path selection and the final evolution stability strategy of the leader enterprise A and follower enterprise B. The Jacobian of the system is as follows.


$$\left[\begin{array}{ll} \begin{array}{l} \left(1-2x)[(P_{A12}-P_{A22})-(1-\alpha )(C_{1}-C_{2}\right)\\ +y(P_{A11}-P_{A21})-y(P_{A12}-P_{A22})] \end{array} & \;x(1-x)[\left(P_{A11}-P_{A21})-(P_{A12}-P_{A22}\right)]\\ \;y(1-y)[\left(P_{B11}-P_{B12})-(P_{B21}-P_{B22}\right)] & \begin{array}{l} \left(1-2y)[(P_{B21}-P_{B22})-(1-\alpha )(C_{1}-C_{2}\right)\\ +x(P_{B11}-P_{B12})-x(P_{B21}-P_{B22})] \end{array} \end{array}\right]\;\mathrm{or}$$

$$\left[\begin{array}{cc} \begin{array}{l} (1-2x)[(P_{A12}-P_{A22})-(C_{1}-C_{2})-\beta \\ \left(k_{1}-k_{2})+y(P_{A11}-P_{A21})-y(P_{A12}-P_{A22})\right] \end{array} & \begin{array}{l} x(1-x)[\left(P_{A11}-P_{A21})-(P_{A12}-P_{A22}\right)] \end{array}\\ y(1-y)[\left(P_{B11}-P_{B12})-(P_{B21}-P_{B22}\right)] & \begin{array}{l} (1-2y)[(P_{B21}-P_{B22})-(C_{1}-C_{2})-\beta \\ \left(k_{3}-k_{4})+x(P_{B11}-P_{B12})-x(P_{B21}-P_{B22})\right] \end{array} \end{array}\right]$$

The determinant and trace values of jacobian matrix at different equilibrium points of the system are shown in the following Table 2.

**Table 2:** The determinant and trace values of jacobian matrix at different equilibrium points.

Equilibrium	Determinant	Trace
*O*(0,0)	$\begin{array}{l} -[\left(P_{A12}-P_{A22})-(1-\alpha )(C_{1}-C_{2}\right)]\\ {}*[\left(P_{B21}-P_{B22})-(1-\alpha )(C_{1}-C_{2}\right)] \end{array}$ or $\begin{array}{l} \left[\left(P_{A12}-P_{A22})-(C_{1}-C_{2})-\beta (k_{1}-k_{2}\right)\right]\\ {}*[\left(P_{B21}-P_{B22})-(C_{1}-C_{2})-\beta (k_{3}-k_{4}\right)] \end{array}$	$\begin{array}{l} {}[\left(P_{A12}-P_{A22})-(1-\alpha )(C_{1}-C_{2}\right)]+\\ \left[\left(P_{B21}-P_{B22})-(1-\alpha )(C_{1}-C_{2}\right)\right] \end{array}$ or $\begin{array}{l} \left[\left(P_{A12}-P_{A22})-(C_{1}-C_{2})-\beta (k_{1}-k_{2}\right)\right]\\ +[\left(P_{B21}-P_{B22})-(C_{1}-C_{2})-\beta (k_{3}-k_{4}\right)] \end{array}$
*U*(1,0)	$\begin{array}{l} -[\left(P_{A12}-P_{A22})-(1-\alpha )(C_{1}-C_{2}\right)]\\ {}*[\left(P_{B21}-P_{B22})-(1-\alpha )(C_{1}-C_{2}\right)] \end{array}$ or $\begin{array}{l} -[\left(P_{A12}-P_{A22})-(C_{1}-C_{2})-\beta (k_{1}-k_{2}\right)]\\ {}*[\left(P_{B11}-P_{B12})-(C_{1}-C_{2})-\beta (k_{3}-k_{4}\right)] \end{array}$	$\begin{array}{l} -[\left(P_{A12}-P_{A22})-(1-\alpha )(C_{1}-C_{2}\right)]+\\ \left[\left(P_{B21}-P_{B22})-(1-\alpha )(C_{1}-C_{2}\right)\right] \end{array}$ or $\begin{array}{l} -[\left(P_{A12}-P_{A22})-(C_{1}-C_{2})-\beta (k_{1}-k_{2}\right)]\\ +[\left(P_{B11}-P_{B12})-(C_{1}-C_{2})-\beta (k_{3}-k_{4}\right)] \end{array}$
*V*(0,1)	$\begin{array}{l} -[\left(P_{A11}-P_{A21})-(1-\alpha )(C_{1}-C_{2}\right)]\\ {}*[\left(P_{B21}-P_{B22})-(1-\alpha )(C_{1}-C_{2}\right)] \end{array}$ or $\begin{array}{l} -[\left(P_{A11}-P_{A21})-(C_{1}-C_{2})-\beta (k_{1}-k_{2}\right)]\\ {}*[\left(P_{B21}-P_{B22})-(C_{1}-C_{2})-\beta (k_{3}-k_{4}\right)] \end{array}$	$\begin{array}{l} \left[\left(P_{A11}-P_{A21})-(1-\alpha )(C_{1}-C_{2}\right)\right]\\ -[\left(P_{B21}-P_{B22})-(1-\alpha )(C_{1}-C_{2}\right)] \end{array}$ or $\begin{array}{l} \left[\left(P_{A11}-P_{A21})-(C_{1}-C_{2})-\beta (k_{1}-k_{2}\right)\right]\\ -[\left(P_{B21}-P_{B22})-(C_{1}-C_{2})-\beta (k_{3}-k_{4}\right)] \end{array}$
*W*(1,1)	$\begin{array}{l} \left[\left(P_{A11}-P_{A21})-(1-\alpha )(C_{1}-C_{2}\right)\right]\\ {}*[\left(P_{B11}-P_{B12})-(1-\alpha )(C_{1}-C_{2}\right)] \end{array}$ or $\begin{array}{l} \left[\left(P_{A11}-P_{A21})-(C_{1}-C_{2})-\beta (k_{1}-k_{2}\right)\right]\\ {}*[\left(P_{B11}-P_{B12})-(C_{1}-C_{2})-\beta (k_{3}-k_{4}\right)] \end{array}$	$\begin{array}{l} -[\left(P_{A11}-P_{A21})-(1-\alpha )(C_{1}-C_{2}\right)]\\ -[\left(P_{B21}-P_{B22})-(1-\alpha )(C_{1}-C_{2}\right)] \end{array}$ or $\begin{array}{l} -[\left(P_{A12}-P_{A22})-(C_{1}-C_{2})-\beta (k_{1}-k_{2}\right)]\\ -[\left(P_{B21}-P_{B22})-(C_{1}-C_{2})-\beta (k_{3}-k_{4}\right)] \end{array}$

When the local stable point satisfies the conditions that the determinant of jacobian matrix is positive and the trace of Jacobian matrix is negative, it is the stable evolution strategy of evolutionary game model. Local stability analysis of the system is shown in Table 3.

**Table 3:** Local stability analysis of the system.

Equilibrium	D(J)	T(J)	Conclusion	D(J)	T(J)	Conclusion	D(J)	T(J)	Conclusion
	Condition Ⅰ			Condition Ⅱ			Condition Ⅲ		
*O*(0,0)	+	-	ESS	+	+	Instability Point	+	+	Instability Point
*U*(1,0)	-	+	Saddle Point	+	-	ESS	-	+	Saddle Point
*V*(0,1)	-	+	Saddle Point	Instability Point	Instability Point	Instability Point	-	+	Saddle Point
*W*(1,1)	+	+	Instability Point	+	+	Instability Point	+	-	ESS
**Equilibrium**	**Condition Ⅳ**			**Condition Ⅴ**			**Condition Ⅵ**		
*O*(0,0)	+	-	ESS	+	+	Instability Point	+	+	Instability Point
*U*(1,0)	-	+	Saddle Point	Instability Point	Instability Point	Instability Point	-	+	Saddle Point
*V*(0,1)	-	+	Saddle Point	+	-	ESS	-	+	Saddle Point
*W*(1,1)	+	+	Instability Point	+	+	Instability Point	+	-	ESS

### 5.2. Evolutionary stability strategy analysis

**Fig 4:**
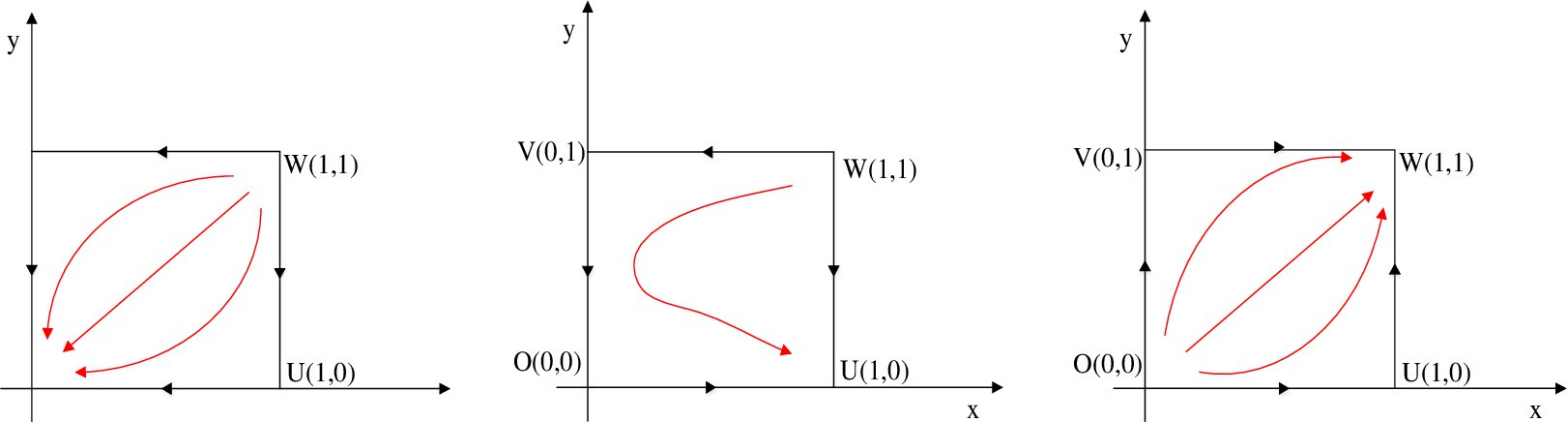
The evolution phase diagram of manufacturing enterprise path selection under the government subsidy for green technology innovation (Ⅰ), (Ⅱ) and (Ⅲ).

**Fig 5:**
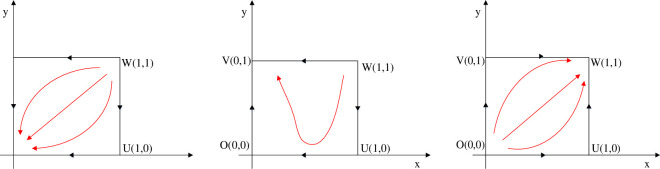
The evolution phase diagram of manufacturing enterprise path selection under the carbon tax(Ⅳ), (Ⅴ) and (Ⅵ).

According to the discriminant method of local stability of Jacobian matrix, the equilibrium point and local stability analysis of the system in the Table 3 are analyzed. The conclusion is as follows and the phase diagram is shown in the Figs 4 and 5 below.

Conclusion (1) When $\alpha <1-\left(P_{A12}-P_{A22}\right)/\left(C_{1}-C_{2}\right)$ (Condition Ⅰ), the local stable point *O*(0,0) is the stable strategy of the system evolution, *U*(1,0) and *V*(0,1) are the saddle points, and *W*(1,1) is the unstable point. When the government subsidy rate for green technology innovation meets condition Ⅰ, no matter what the initial state of the system is, the system will converge to the evolutionary stable strategy *O*(0,0). In other words, all manufacturing enterprises choose the path of EGTI, and the level of social benefit at this time is the lowest. More specifically, the input cost of manufacturing enterprises choosing the path of IIGT is high, while the government subsidy for green technology innovation is pretty low. Although the profit of the enterprise is maintained temporarily, the marginal cost of unit product under this path is still too high and the pollution is serious due to the small amount of subsidy, which is not conducive to the long-term development of the manufacturing enterprise and the requirements of low-carbon and green development of the society. Owing to avoid the overall profit decline, manufacturing enterprises choose the path of EGTI with lower cost. Therefore, the proportion of enterprises that choose the path of IIGT will gradually decrease until all enterprises choose the path of EGTI after a long-term game.

Conclusion (2) When $\{\begin{array}{c} \alpha >1-\left(P_{A12}-P_{A22}\right)/\left(C_{1}-C_{2}\right)\\ \alpha <1-\left(P_{B11}-P_{B12}\right)/\left(C_{1}-C_{2}\right) \end{array}$ (Condition Ⅱ), the local stable point *U*(1,0) is the stable strategy of the system evolution, *O*(0,0), *V*(0,1) and *W*(1,1) are the unstable points. The system will converge to the evolutionary stable strategy *U*(1,0) no matter what the initial state of the system is under condition Ⅱ. At this point, the leading manufacturing enterprise occupies favorable conditions in the market, and the actual marginal cost of unit product will be reduced on the basis of government subsidies for green technology innovation input. In addition, the product quality obtained through IIGT is greatly improved, and it will occupy a more favorable position in the market. Therefore, the leading manufacturing enterprise A will choose the path of IIGT. In view of the limited market share and high capital of independent R&D, the follower enterprise B will continue to choose the low-cost green technology innovation path of EGTI if the government subsidy for green technology innovation fails to reach a certain standard. It is worth noting that the path of EGTI will far lag behind the benefits gained from IIGT of green technology, and the follower enterprise B may lose market share after a long time.

Conclusion (3) When $\alpha >1-\left(P_{B11}-P_{B12}\right)/\left(C_{1}-C_{2}\right)$ (Condition Ⅲ), the local stable point *W*(1,1) is the stable strategy of the system evolution, *U*(1,0) and *V*(0,1) are the saddle points, and *O*(0,0) is the unstable point. Under condition Ⅲ, the minimum additional net income obtained by the game players in the system through the path of IIGT is positive. The system will converge to the evolutionary stable strategy *W*(1,1) no matter what the initial state of the system is. That is to say, all manufacturing enterprises choose the path of IIGT, and the level of social benefit at this time is the lowest. In particular, the government encourages manufacturing enterprises to carry out independent green technology innovation and gives them large subsidies for green technology innovation. Driven by economic interests, enterprises choose the path of IIGT to reduce the marginal cost of unit products and improve profits after many games.

Conclusion (4) When $\beta >\left[\left(C_{1}-C_{2})-(P_{B21}-P_{B22}\right)\right]/\left(k_{4}-k_{3}\right)$ (Condition Ⅳ), the local stable point *O*(0,0) is the stable strategy of the system evolution, *U*(1,0) and *V*(0,1) are the saddle points, and *W*(1,1) is the unstable point. Under condition Ⅳ, the system will converge to the evolutionary stable strategy *O*(0,0) no matter what the initial state of the system is, and all manufacturing enterprises choose the path of EGTI. In this case, the carbon tax rate levied by the government is relatively low, and the carbon tax paid by manufacturing enterprises through the path of EGTI is far less than the cost of independent green technology innovation. Considering the overall profits of manufacturing enterprises, they are more willing to bear a small part of the carbon tax cost, rather than more investment of independent R&D green technology innovation. Therefore, the game players will choose the path of EGTI with lower cost of green technology innovation after a long-term evolutionary game. Finally, the proportion of enterprises that choose the path of IIGT will gradually decrease until all enterprises choose the path of EGTI, so as to obtain greater profits.

Conclusion (5) When $\{\begin{array}{c} \beta >\left[\left(C_{1}-C_{2})-(P_{B21}-P_{B22}\right)\right]/\left(k_{4}-k_{3}\right)\\ \beta <\left[\left(C_{1}-C_{2})-(P_{A11}-P_{B21}\right)\right]/\left(k_{2}-k_{1}\right) \end{array}$ (Condition Ⅴ), the local stable point *V*(0,1) is the stable strategy of the system evolution, *O*(0,0), *U*(1,0) and *W*(1,1) are the unstable points. When the government increases the carbon tax rate to meet the condition Ⅴ, the system will eventually converge to the evolutionary stable strategy *V*(0,1). Compared with the high investment cost of IIGT, the optimal choice of is the manufacture A to pay carbon tax, and the manufacture A will still choose the path of EGTI in the circumstances. However, the carbon tax paid by the follower enterprise B is greater than the investment cost of IIGT in this case. If the follower enterprise B continues to choose the path of EGTI, it will not be able to effectively reduce carbon emissions, which will eventually lead to the decline of the overall profit of the follower enterprise B. As a follower, the follower enterprise B will instead choose to carry out IIGT.

Conclusion (6) When $\beta >\left[\left(C_{1}-C_{2})-(P_{A11}-P_{B21}\right)\right]/\left(k_{2}-k_{1}\right)$ (Condition Ⅵ), the local stable point *W*(1,1) is the stable strategy of the system evolution, *U*(1,0) and *V*(0,1) are the saddle points, and *O*(0,0) is the unstable point. Under condition Ⅵ, the minimum additional net income obtained by the game players in the system through the path of IIGT is positive. The system will converge to the evolutionary stable strategy *W*(1,1) no matter what the initial state of the system is. That is to say, all manufacturing enterprises choose the path of IIGT. On this occasion, the government increased the carbon tax rate to control the carbon emission of manufacturing enterprises. The carbon tax amount paid by the leading leader enterprise A is also greater than the investment cost of technology introduction. If it continues to choose the path of EGTI, carbon emissions cannot be effectively reduced. Therefore, the leader enterprise A chooses to reduce carbon emissions and carbon tax payment through IIGT, so as to improve profits.

## 6. Simulations and analysis

**Fig 6:**
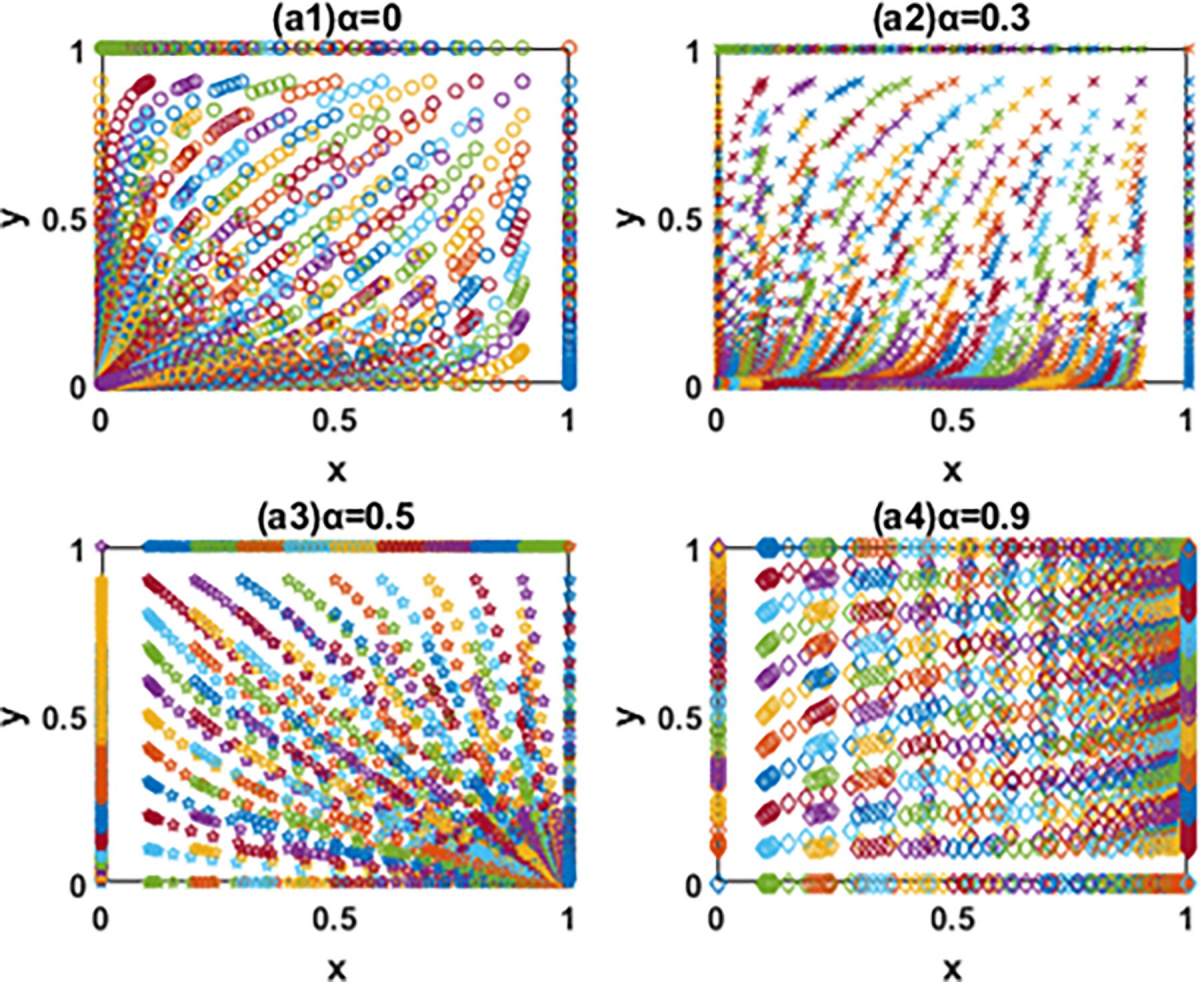
Effects of α on the path selection of system.

**Fig 7:**
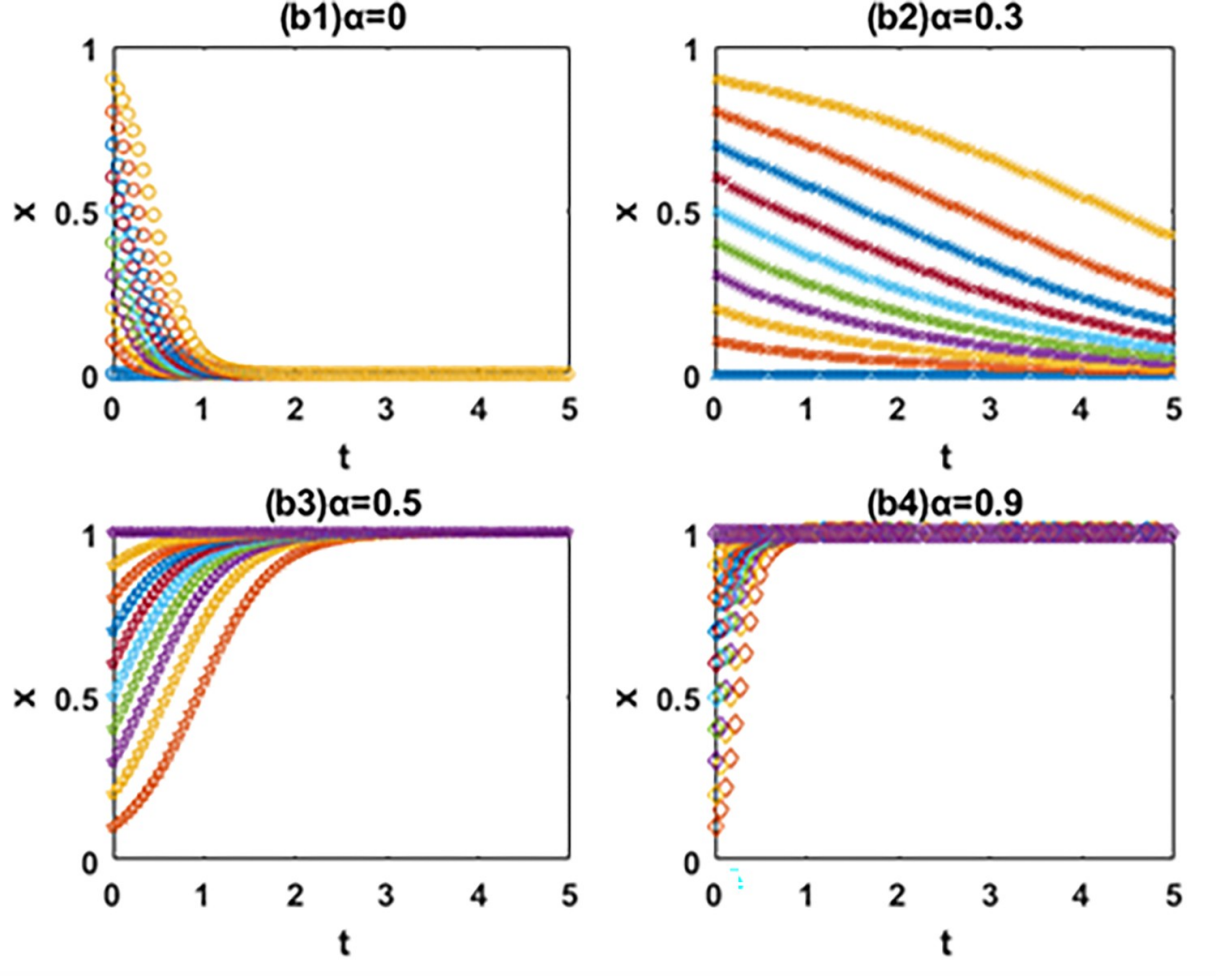
Effects of α on the path selection of the leader enterprise A.

**Fig 8:**
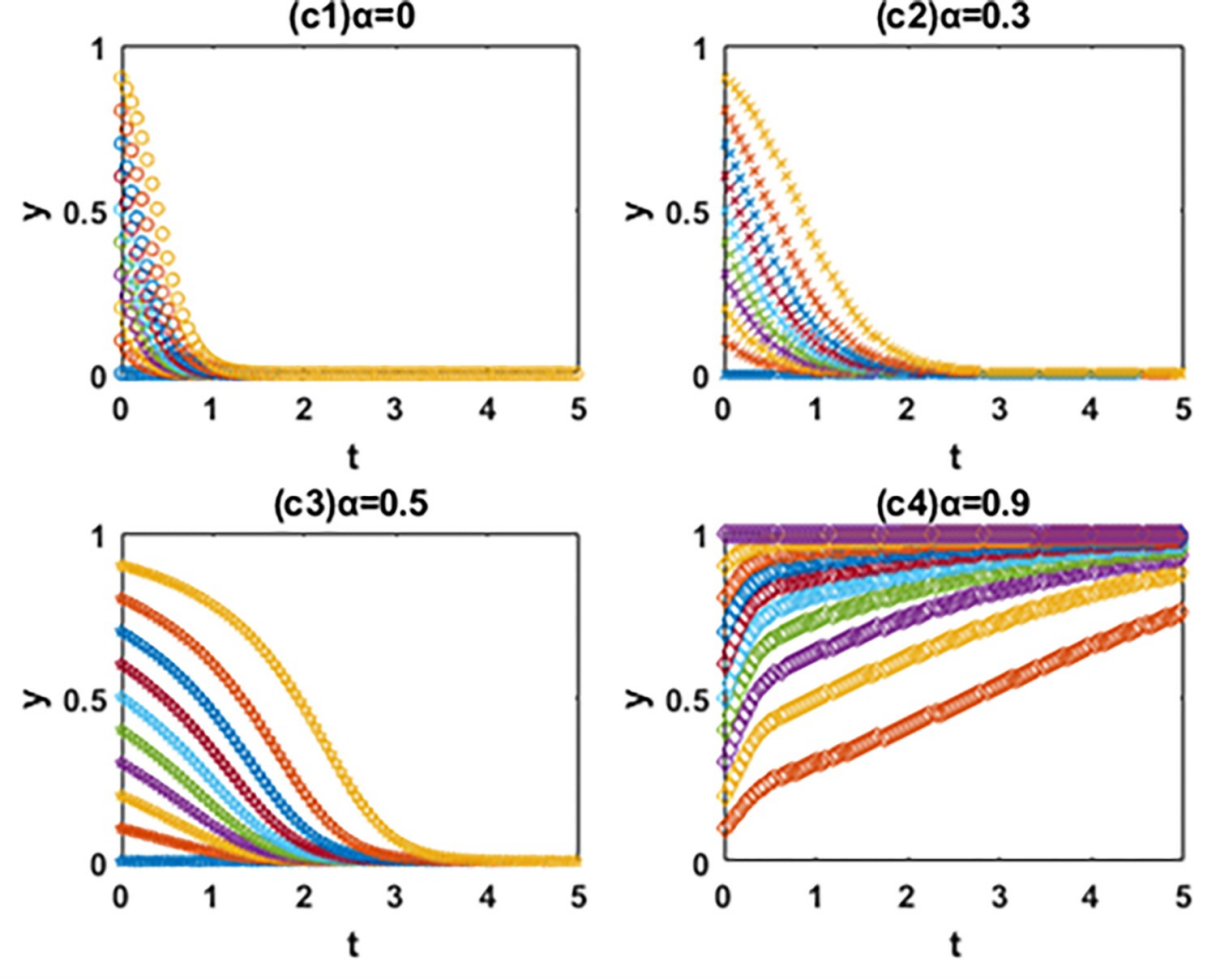
Effects of α on the path selection of the follower enterprise B.

**Fig 9:**
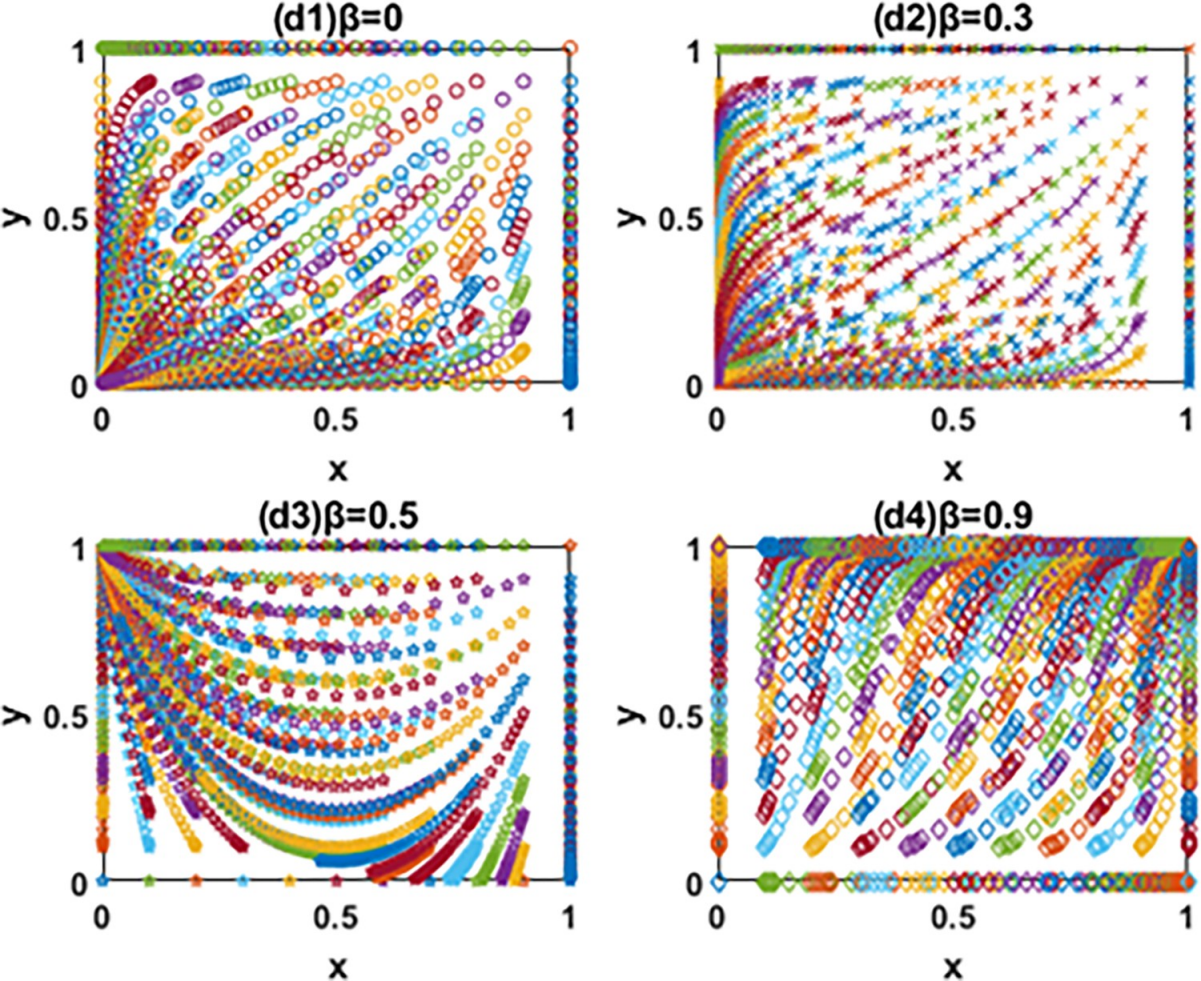
Effects of β on the path selection of system.

**Fig 10:**
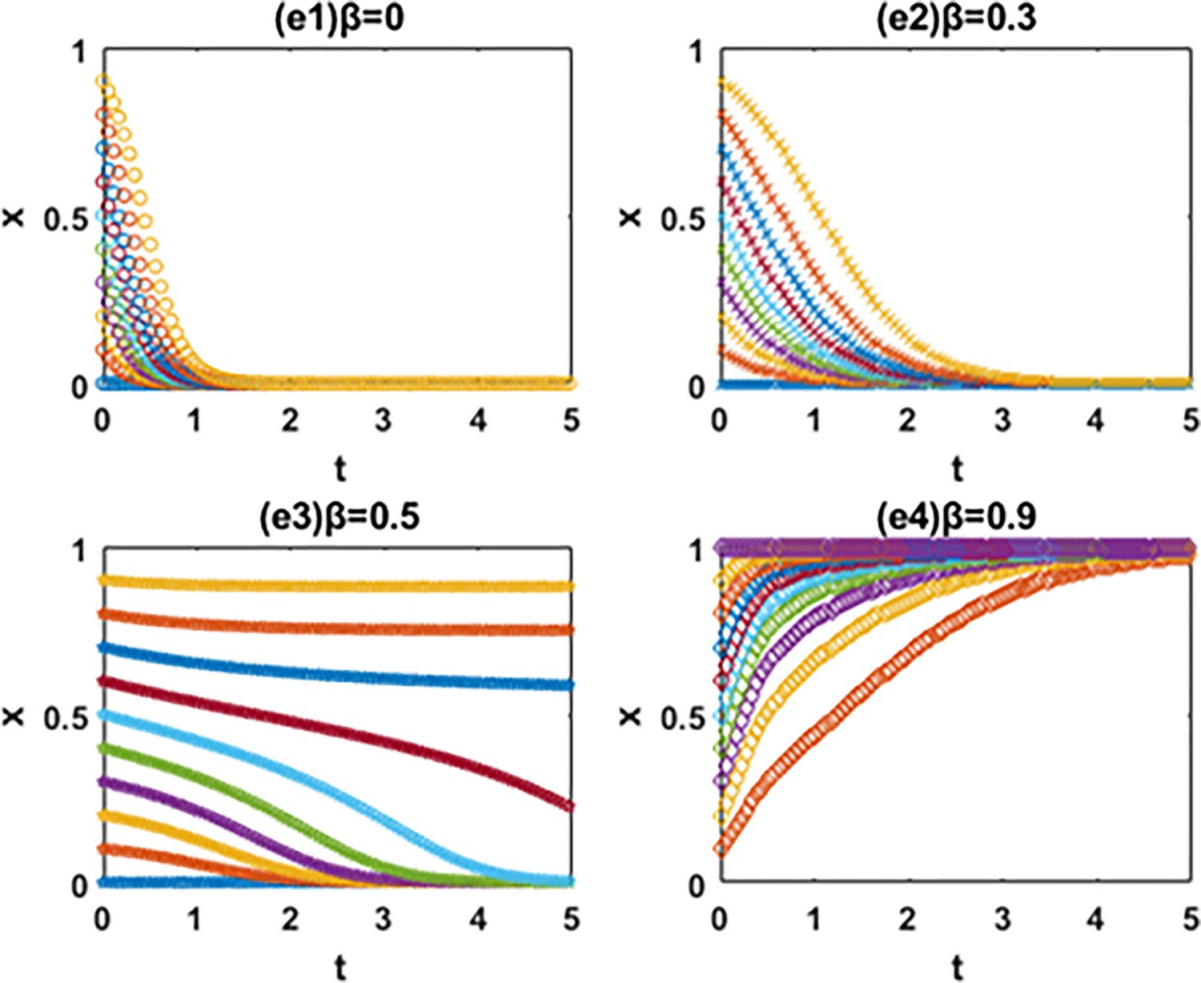
Effects of β on the path selection of the leader enterprise A.

In this paper, the evolutionary game theory is used to analyze the influence of government subsidies for green innovation and carbon tax on the choice of green technology innovation path of manufacturing enterprises. In order to further study the evolution mechanism of the system, this paper further simulates the evolution process of the system by using MATLAB software according to the constraints and replication dynamic equations. The influence of government subsidies for green innovation and carbon tax on the decision-making process and results of green technology innovation path selection of manufacturing enterprises is analyzed by comparing the value of changing parameters. It is assumed that x and y are the initial proportion of the leader enterprise A and follower enterprise B choosing the path of IIGT and EGTI. According to the actual situation and basic assumptions of the model, the values of simulation parameters are set respectively: *a* = 60, *b* = 1, *α* = 1, *β* = 1, *C*_1_ = 55, *C*_2_ = 53, *C*_3_ = 56, *C*_4_ = 54, *k*_1_ = 2, *k*_2_ = 5, *k*_3_ = 1 and *k*_4_ = 4. The initial proportion of the leader enterprise A and follower enterprise B that choose the path of IIGT and EGTI is set to change isometric from 0 to 1 respectively. The evolution paths of system, x and y are depicted when the subsidy rate of green technology innovation input is 0 (initial state), 0.1 (condition I), 0.5 (condition Ⅱ) and 0.9 (condition III) in Figs 5–7, and when the carbon tax rate is 0 (initial state), 0.3 (condition Ⅳ), 0.6 (condition Ⅴ) and 0.9 (condition Ⅵ) in Figs 8–10 respectively.

### 6.1. Numerical simulation of the system evolution under the government subsidies for green innovation

**(1) Effects of α on the path selection of system.** From Fig 6, first, the system will eventually evolve to the coordinate point (0,0) and all enterprises choose the path of EGTI when α = 0 and 0.1. Given that the low government subsidy rate cannot make up for the cost of IIGT, any enterprise can not be stimulated to implement IIGT. After the long-term game, the proportion of enterprises that choose the path of IIGT in the system will gradually reduce until all enterprises choose the path of EGTI. Second, when α = 0.5 and the initial state is in the lower right region, the system converges to the coordinate point (1,0). Under this condition, the system will eventually evolve to a stable state where the leader enterprise A and follower enterprise B chooses the path of IIGT and EGTI respectively. Third, the system will eventually evolve to the coordinate point (1,1) and all enterprises choose the path of IIGT when α = 0.9. At this time, the government invested large subsidies for green technology innovation, and enterprises cannot obtain profits with competitive advantages through the EGTI. Therefore, enterprises that have chosen the path of EGTI will give up this path and implement the path of IIGT.

**(2) Effects of α on the path selection of the leader enterprise A.** From Fig 7, the leader enterprise A will choose the path of EGTI when α = 0 or the government has no subsidy for green technology innovation. When α = 0.1, the evolution speed of the leader enterprise A to choose the path of IIGT increases to a certain extent, but it is not obvious. The results show that when the government subsidy rate is small, the incentive of environmental regulation on the independent innovation of leading enterprises can only play a role in a certain range. When α = 0, the leader enterprise A will choose the path of IIGT. When α = 0.9, the leader enterprise A evolves rapidly to choose the path of IIGT.

**(3) Effects of α on the path selection of the follower enterprise B.** From Fig 8, when α = 0, 0.1 and 0.5, it means that the government has no subsidy for green technology innovation or the subsidy rate is not high enough, the follower enterprise B will eventually choose the path of EGTI. It is worth noting that when the value of α changes from 0 to 0.5, the evolution speed and the probability of the follower enterprise B to the choose the path of EGTI gradually slows down and gets smaller. When α = 0.9, the follower enterprise B will choose the path of IIGT.

In general, the leader enterprise A is in a leading position in the market with abundant funds and a large market share. When the government subsidy for green technology innovation is relatively small, the leader enterprise A will actively choose the path of IIGT earlier than the follower enterprise B. It aims to reduce the marginal cost of products, expand the market, increase revenue and ensure the long-term development of the enterprise [61]. The follower follower enterprise B will continue to adopt the path of EGTI at the beginning and choose the path of IIGT when the government subsidy for green technology innovation reaches a certain level. The reason is that compared with IIGT, both players of the game will choose the path of EGTI with less time and cost at the beginning [62]. When the government provides subsidies for green technology innovation, the leader enterprise A is vulnerable to subsidies and makes profits through IIGT based on its market position and financial resources. However, due to its limited economic strength, the follower enterprise B was later promoted by the government subsidies for green technology innovation [63].

### 6.2. Numerical simulation of the system evolution under the carbon tax

**(1) Effects of β on the path selection of system.** From Fig 9, the system will eventually evolve to the coordinate point (0,0) and all enterprises choose the path of EGTI when β = 0 and 0.3. At this time, carbon taxes are too low to motivate the manufacturers to focus on green innovation. Manufacturing enterprises choosing the path of IIGT will not increase profits, but reduce corporate profits. After the long-term game, the proportion of enterprises that choose the path of IIGT in the system will gradually reduce until all enterprises choose the path of EGTI. When β = 0.6, the system will eventually evolve to a stable state where the leader enterprise A and follower enterprise B chooses the path of EGTI and IIGT respectively. When β = 0.9, the system will eventually evolve to the coordinate point (1,1) and all enterprises choose the path of IIGT. At this time, the government’s carbon tax is far greater than the investment cost of IIGT. The game players cannot gain benefits with competitive advantages through the path of EGTI. After a long-term game, the proportion of enterprises choosing the path of EGTI in the system will gradually decrease until all enterprises choose the path of IIGT.

**(2) Effects of β on the path selection of the leader enterprise A.** From Fig 10, when β = 0 and 0.3, the government do not levy carbon taxes or carbon taxes are not high enough, and the leader enterprise A will choose the path of EGTI. When β = 0.6, the evolution speed of the leader enterprise A to choose the path of IIGT increases to a certain extent. This means that the probability of the leader enterprise A choosing the path of EGTI will gradually decrease, but the evolutionary path does not converge. In this case, the collection of government’s carbon tax cannot give full play to the incentive role of leading enterprises to implement independent innovation. When β = 0.9, the leader enterprise A will ultimately choose the path of IIGT.

**Fig 11:**
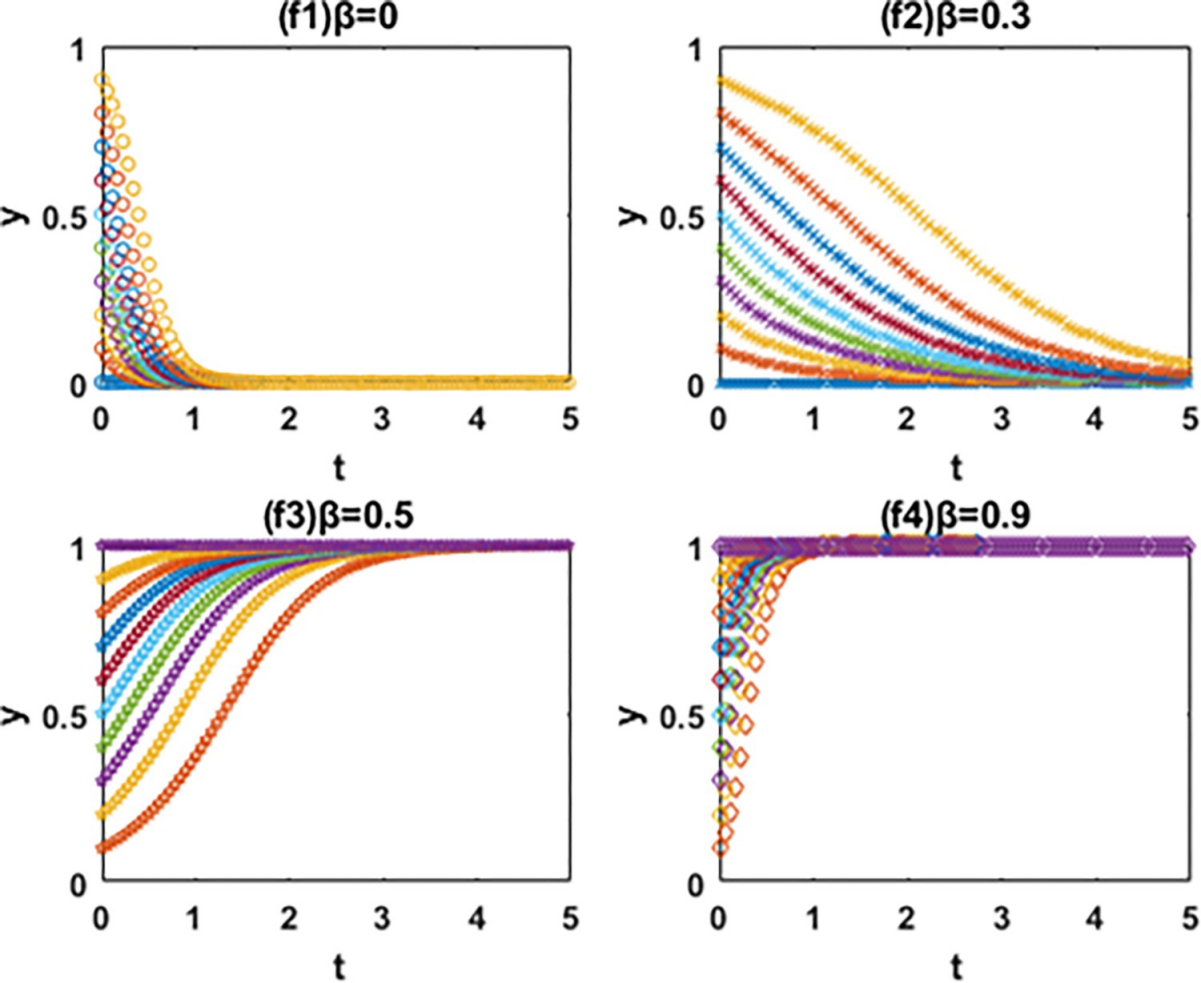
Effects of β on the path selection of the follower enterprise B.

**(3) Effects of β on the path selection of the follower enterprise B.** From Fig 11, when β = 0 and 0.3, the government do not levy carbon taxes or carbon taxes are not high enough, and the follower enterprise B will choose the path of EGTI. when β = 0.6 and 0.9, the follower enterprise B will choose the path of IIGT. When the value of β changes from 0.6 to 0.9, the evolution speed and the probability of the follower enterprise B to the choose the path of IIGT gradually gets faster and bigger.

In particular, when the carbon tax rate is relatively small, the leader enterprise A will continue to adopt the path of EGTI at the beginning and choose the path of IIGT when the carbon tax rate reaches a certain level. And the follower enterprise B will actively choose the path of IIGT earlier than the leader enterprise A. However, when the government begins to impose a carbon tax on enterprises, the leader enterprise A was later affected by the carbon tax costs based on its own market status and financial resources. The carbon tax amount paid by the follower enterprise B will exceed the input cost of IIGT earlier [64].

On the whole, the path selection results of manufacturing enterprises are closely related to government subsidies and carbon tax rate, and the decision-making processes among manufacturing enterprises influence each other. Besides, the government subsidies for green innovation and carbon tax rate have an incentive effect on the evolution speed of the leader enterprise A and follower enterprise B to the direction of IIGT [65], and the greater the subsidy and carbon tax rate, the more obvious the incentive effect. The incentive effect of government subsidies on the leader A to choose the path of IIGT is earlier than that of the follower enterprise B. However, the incentive effect of carbon tax rate on the follower enterprise B to choose the path of IIGT is earlier than that of the leader A.

## 7. Conclusions and implications

### 7.1. Conclusions

Environmental regulation and technology innovation have always been the focus of academic research. However, the research on how to carry out green technology innovation and achieve the target of energy conservation and emission reduction in China’s manufacturing enterprises is still at the theoretical level with different conclusions and relatively scattered. With the improvement of China’s environmental regulation system, in the context of serious heterogeneity of green technology innovation level at various levels of manufacturing industry, it is necessary to make a systematic and in-depth study on the impact of environmental regulation on the green technology innovation path of manufacturing enterprises, so as to provide effective reference for the government to promote the green transformation and upgrading of manufacturing enterprises.This paper firstly puts forward two types of green technology innovation paths considering the reality of China, and analyzes the heterogeneous influence mechanism of environmental regulation on the path of IIGT and EGTI. Secondly, based on evolutionary game theory, a two-agent game model of environmental regulation on the green technology innovation path selection of manufacturing enterprises was constructed, and then the influence of two types of environmental regulation tools on the choice of green technology innovation path of China’s manufacturing enterprises was studied from two aspects of the government subsidies for green innovation and carbon tax rate. Finally, this paper simulates the influence of various parameters on the evolution path and speed of green technology innovation path selection of the system, leader enterprise A and follower enterprise B under different circumstances by numerical simulation.

The conclusions are drawn as follows:

1. When the government does not implement environmental regulation, the system, leader enterprise A and follower enterprise B will eventually choose the path of EGTI after a long-term evolution process. When the government implement environmental regulation, the result changes in the opposite direction. After a long-term evolution process, the system, leader enterprise A and follower enterprise B will eventually choose the path of IIGT.
2. There are differences in the influence of different types of government regulation on green technology innovation path selection of manufacturing enterprises. Both the increase of the government subsidies for green innovation and carbon tax rate can encourage manufacturing enterprises to choose the path of IIGT with greater possibility, and the decision-making process of path selection among enterprises is closely related.
3. When the government adopts the regulation tools of subsidies for green innovation, the leader enterprise A will actively choose the path of IIGT earlier than the follower enterprise B. However, when the government adopts the regulation tools of carbon tax, the follower enterprise B will actively choose the path of IIGT earlier than the leader enterprise A.

### 7.2. Implications

Government regulation policy is a practical exploration to guide green development through environmental regulation tools, but there is a gap on how environmental regulation affects the path selection of enterprise green technology innovation. Different from the existing literature, this paper analyzes the operation mechanism of green technology innovation path, and provides ideas for how to adjust the decision-making behavior of green technology innovation path selection in manufacturing enterprises under environmental regulation.

The simulation results of this paper have both theoretical and practical value, and the empirical evidence of the above analysis may bring some implications to stimulate green technology innovation of manufacturing enterprises. For example, the government should combine the situation of manufacturing enterprises to implement differentiated environmental regulation policies. First, the government should take effective economic incentives instead of administrative penalties,increase support for green R&D, and provide sufficient time, information and green innovation-friendly policies for enterprises to implement green technological innovation. Second, while providing tax incentives and institutional guarantees for enterprises to carry out R&D activities, the government should also consider the scale effect of enterprise size on green innovation. In particular, the government should provide more financing channels and financial subsidies for the leading large-scale manufacturing enterprises, and provide low-guarantee mortgage loans for the following small and medium-sized enterprises to help enterprises implement green technological innovation. Third, since the carbon tax rate has a more significant impact on the speed of enterprise evolution, and even changes the evolution direction of the final state of enterprises, the imposition of carbon tax is more suitable for China’s current economic development. The government should pay close attention to the development of China’s manufacturing industry and market, formulate environmental policies in line with the development status of China’s manufacturing industry, moderately increase the level of subsidies and carbon tax, and encourage manufacturing enterprises to carry out breakthrough green innovation. Fourthly, the government should determine the optimal subsidy rate or carbon tax rate according to the differences in production costs of enterprises, and take the lead in promoting the rapid growth of green technologies among enterprises with cost advantages in the industry, thus promoting the healthy development of the entire industry and market.

Although the relevant viewpoints proposed in this paper are supported by theoretical and practical data, there are still some areas to be improved due to the time and level of research. For example, international economic and institutional factors are not considered in the given research hypothesis and model construction, which is limited to the local context of China. In fact, the choice of green technology innovation path by China’s manufacturing enterprises is largely influenced by international related factors. Future research should focus on more influencing factors. In addition, due to the limitation of game model, the next research can focus on the multi-agent game behavior instead of the finite parameters under two game players.
